# Microsphere-based antibody assays for human parvovirus B19V, CMV and *T. gondii*

**DOI:** 10.1186/s12879-015-1194-3

**Published:** 2016-01-08

**Authors:** Yilin Wang, Lea Hedman, Maria F. Perdomo, Amal Elfaitouri, Agnes Bölin-Wiener, Arun Kumar, Maija Lappalainen, Maria Söderlund-Venermo, Jonas Blomberg, Klaus Hedman

**Affiliations:** 1 Virology, University of Helsinki, Haartmaninkatu 3, FI-00290 Helsinki, Finland; 2 Helsinki University Hospital, Helsinki, Finland; 3 Section of Clinical Virology, Department of Medical Sciences, Uppsala University and Uppsala University Hospital, Uppsala, Sweden; 4 Present address: T-Cell Platform, Translational Medicine, Novartis Vaccines (a GSK company), Siena, Italy

**Keywords:** Microsphere-based suspension immuno assay, Singleplex and multiplex, B19V, CMV, *T. gondii*, Pregnancy

## Abstract

**Background:**

Human parvovirus B19 (B19V), cytomegalovirus (CMV) and *Toxoplasma gondii* (*T. gondii*) may cause intrauterine infections with potentially severe consequences to the fetus. Current serodiagnosis of these infections is based on detection of antibodies most often by EIA and individually for each pathogen. We developed singleplex and multiplex microsphere-based Suspension Immuno Assays (SIAs) for the simultaneous detection of IgG antibodies against B19V, CMV and *T. gondii*.

**Methods:**

We tested the performances of SIAs as compared to in-house and commercial reference assays using serum samples from well-characterized cohorts.

**Results:**

The IgG SIAs for CMV and *T. gondii* showed good concordance with the corresponding Vidas serodiagnostics. The B19V IgG SIA detected IgG in all samples collected >10 days after onset of symptoms and showed high concordance with EIAs (in-house and Biotrin). The serodiagnostics for these three pathogens performed well in multiplex format.

**Conclusions:**

We developed singleplex and multiplex IgG SIAs for the detection of anti-B19V,-CMV and -*T. gondii* antibodies. The SIAs were highly sensitive and specific, and had a wide dynamic range. These components thus should be suitable for construction of a multiplex test for antibody screening during pregnancy.

**Electronic supplementary material:**

The online version of this article (doi:10.1186/s12879-015-1194-3) contains supplementary material, which is available to authorized users.

## Background

Human parvovirus B19 (B19V), cytomegalovirus (CMV) and *Toxoplasma gondii* (*T. gondii*) are ubiquitous pathogens causing diverse clinical manifestations. Their primary infections during pregnancy may lead to severe sequelae. Thus, the serological status of the mother is critically important in counselling and recognition of acute infection [1–3].

The current standards in antibody detection are enzyme-immuno assay (EIA) and chemiluminescence immune assay (CLIA) performed individually for each pathogen; the cost of which has limited its widespread use particularly when related to the overall low incidence of these conditions [4]. Multiplex arrays could be a feasible alternative thanks to their high-throughput performance and efficiency in terms of time and cost as well as sample volume requirement.

We developed Luminex-based singleplex and multiplex microsphere Suspension Immuno Assays (SIAs) to detect IgG antibodies against these three pathogens.

We determined the performances of the SIAs in selected cohort groups (both in singleplex and multiplex format) in comparison to corresponding high-quality in-house or commercial EIAs.

## Methods

The B19V IgG SIA was set up and examined at the University of Helsinki while the CMV and *T. gondii* IgG SIAs were set up at the Uppsala University Hospital. The multiplex IgG SIA for the three pathogens was performed at the University of Helsinki.

### Study populations

B19V IgG SIA: serum samples from 80 children or adults (2 to 58 years of age, median 10 years) with symptomatic B19V infection [1, 5]. These included single samples from 16 subjects and 171 samples from 64 subjects followed up serologically for up to 700 days after primary infection. As control group we included serum samples from 104 constitutionally healthy medical students. The seroprevalences of B19V and several emerging viruses [6–8] have been previously determined with these samples.

CMV and *T. gondii* IgG SIAs: serum samples from 72 subjects; single sera from 64 subjects and two consecutive sera from eight subjects. These samples have been tested for IgGs against *T. gondii* and CMV by the corresponding Vidas enzyme linked fluorescent assays (ELFAs, bioMérieux) and in-house B19V EIA.

For multiplex IgG SIA we used a total of 80 samples as defined above in CMV and *T. gondii* IgG SIAs.

### Coupling of antigens to magnetic/non-magnetic microspheres

The following antigens were coupled with microspheres: for B19V IgG, cloned and purified in-house recombinant VP2 virus-like particles (VLPs) [1, 5, 9]. For CMV IgG, we used purified viral lysate (strain AD 169; Advanced Biotechnology) and for *T. gondii* IgG, purified tachyzoite lysate (RH strain; Advanced Biotechnology). The antigens and conditions for each assay are presented in Table 1.

**Table 1:** Antigens and conditions used in each assay

SIAs	Antigen (Ag)	Ag amount (/coupling)	Serum dilution	Cutoff criterion	Cutoff
B19V VP2 IgG	Recombinant VP2	50 μg	1:20	Mean + 4 SD	Negative < 453 MFI^a^
				Mean + 5 SD	Positive > 532 MFI^a^
CMV IgG	Purified virion lysate	20 μg	1:20	Mean + 4 SD	Negative < 695 MFI^a^
				Mean + 5 SD	Positive > 834 MFI^a^
*T. gondii* IgG	Purified lysate	50 μg	1:20	Mean + 4 SD	Negative < 234 MFI^a^
					Positive > 234 MFI^a^

^a^MFI, median fluorescence intensity

The B19V-magnetic microsphere coupling was performed according to manufacturer’s protocol (“Sample protocol for two step carbodiimide coupling of protein to magnetic microsphere”, Luminex based xMap technology). In each coupling, a total of 1.25 × 10^6^ microsphere (100 μl) were coated with the corresponding antigen/protein followed by blocking with PBS + 50 mM/L Tris + 0.5 mL/L Tween-20 [10]. The coupled microspheres were washed twice with StabilGuard buffer (SG, SurModics) and stored in 1 mL SG at 4 °C in the dark. The antigen concentration was optimized by titration ranging from 200 ug to 0.8 ug per coupling.

The CMV- and *T. gondii*- microsphere couplings were performed using non-magnetic microspheres as described by Elfaitouri et al. [11].

### Internal controls

#### Naked microspheres

To estimate nonspecific binding, each test sample was run together with uncoupled (“naked”) microspheres stored in SG.

### Suspension immunoassays

The B19V SIA was performed following the manufacturer’s protocol (“Sample protocol for indirect antibody capture immunoassay”). The B19V SIA was optimized with serial serum dilutions ranging from 1:5 to 1:320 in phosphate buffered saline with 0.05 % Tween-20 (PBST). The optimal serum dilution is presented in Table 1. Naked microspheres were included as background control. In brief, 50 μl of diluted sera were dispersed in each well of 96-well flat bottom plate. Then the diluted sera were incubated with B19V-coated and naked microspheres for 45 minutes in the dark. After 3 times washing cycles, 50 μl of 2 ~ 4 μg/ml biotinylated protein G (Thermo Scientific) was added in each well. The plate was thoroughly mixed and incubated for 30 minutes in the dark. After 3 times washing cycles, the specific signal was developed by incubation with 2 ~ 4 μg/ml streptavidin conjugated phycoerythrin (R-PE, Life Technologies) in PBST. Each well was resuspended in 120 μl of PBST and read on the Bio-Plex®200 instrument.

The CMV and *T. gondii* SIAs were performed as described [11]. They were also compared with 27 sera against the corresponding Siemens BEPIII IgG tests, with good concordance (courtesy of Bo Albinson, Uppsala; data not shown).

### Multiplex IgG SIA

We tested the B19V, CMV and *T. gondii* IgG SIAs in the multiplex format using 80 sera with known IgG reactivity, and in the same assay conditions as specified for the B19V IgG SIA.

### Reproducibility

In multiplex and singleplex SIAs, the intra-assay variability was calculated with 8 replicates in the same run, and the inter-assay variability with 6 distinct runs.

### Cutoff value determination

The SIAs cutoffs were calculated by the means and standard deviations (SDs) of test values. For B19V IgG SIA, the cutoff was defined with 72 sera confirmed to be B19V seronegative by both Biotrin’s and in-house B19V IgG and IgM EIAs. The sera were from children with expiratory wheezing, studied earlier for human bocavirus 1 [7]. The CMV and *T. gondii* cut-offs were established with separate sets of 60 sera each. These samples were IgG seronegative as confirmed by the Abott assays (Table 1).

### Statistical analysis

We used two-way contingency table analysis in ‘VassarStats’ for the calculation of kappa value, sensitivity, and specificity. Borderline values in reference assays were excluded from the calculations. The agreement between SIA and EIA was evaluated by kappa values and defined as: poor (<0.20), fair (0.21–0.40), moderate (0.41–0.60), good (0.61–0.80), and very good (0.81–1.00) [12]. Pearson correlation coeffients (R^2^) were calculated by GraphPad Prism 6 to determine the correlation of results between singleplex and multiplex IgG SIAs.

### Cost calculation

The net cost was calculated by the sum of all the reagents’ costs (see Additional File 1).

### Ethical approval

The Helsinki University Hospital Ethics Committee approved the use of all clinical samples included in this study (Dnro 553/E6/2001, § 66, 13.4.2011). The control serum samples in B19V SIA study were obtained from the medical students with informed consent. All other samples in this study were taken as part of standard care and were analyzed anonymously.

## Results

### B19V IgG SIA

We validated the B19V IgG SIA using samples from **a)** symptomatic B19V patients and **b)** constitutionally healthy medical students.

**Fig. 1:**
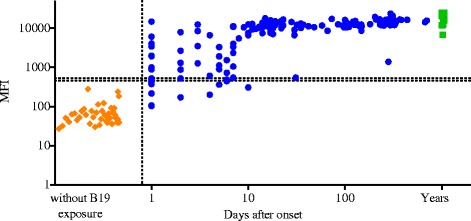
IgG response to B19V VP2. Represented are the IgG seroresponses to B19V VP2 (circle in blue) in 65 subjects with symptomatic B19V infection. The controls were asymptomatic individuals of whom 63 were IgG positive by B19V VP2 (box dot in green). Shown with diamond are the IgG seronegative healthy individuals from the same control cohort. *Y-axis* shows median fluorescence intensity (MFI) by SIA and *x-axis* shows days after onset. The vertical dashed line represents day 0 after onset of symptoms. The horizontal dashed lines depict the B19V IgG SIA cutoff values

**Fig. 2:**
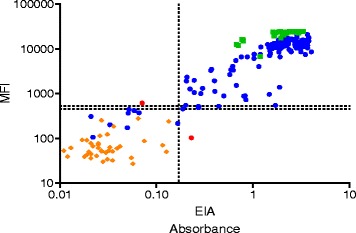
Comparison of B19V IgG results by SIA and EIA. Represented is the comparison of IgG levels determined by SIA (*y-axis*) and EIA (*x-axis*) in sera from patients with symptomatic B19V-infection (circle in blue). Circle dots in red show two discrepancies between SIA and EIA. The controls were asymptomatic individuals of whom 63 were IgG positive by B19V VP2 (box dot in green). Shown with diamond are the IgG seronegative healthy individuals from the same control cohort. The horizontal and vertical dashed lines depict the B19V IgG SIA and EIA cutoff values, respectively

From the symptomatic B19V individuals we tested 143 serum samples of which 16 corresponded to single sera from 16 subjects and 127 follow-up sera from 49 subjects. We found that 90 % (129/143) of the samples were IgG positive and 4 had borderline results. All samples collected >10 days after onset of symptoms showed a 100 % assay concordance with B19V IgG in-house EIA (Fig. 1). The four borderline samples had been collected within 6 days of onset; three of them were weakly positive in EIA (absorbance value: 0.2–0.4), and one was negative. Overall, two discrepancies were found: one, SIA+ EIA-, was collected on day 4 and the other, SIA- EIA+, on day 1 after onset (Fig. 2).

In the cohort of healthy individuals, we found a seropositivity of 60 % in B19V IgG SIA. This finding was in full agreement with that of EIA (Fig. 2).

Compared to in-house EIA, the B19V IgG SIA showed 98 % in both sensitivity and specificity, with very good agreement (kappa coefficient 95 %; CI: 0.95 ~ 1). The intra-assay and inter-assay variability of B19V IgG SIA were 3 ~ 8 % and 1 ~ 12 %.

### CMV and *T. gondii* IgG SIAs

**Fig. 3:**
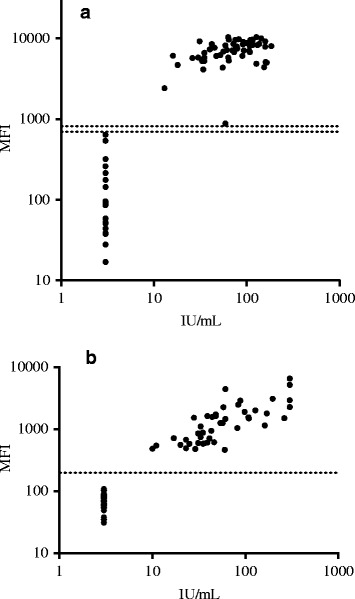
Comparison of anti-CMV and anti-*T. gondii* IgG seroresponses by SIA and ELFA. Represented are (**a**) CMV IgG and (**b**) *T. gondii* IgG responses by SIAs (*y-axis*) or Vidas ELFA (*x-axis*) from 72 individuals. Horizontal dot lines indicate the cutoffs between negative and positive samples

We used samples with known reactivity against CMV and *T. gondii* in commercial assays. In concordance with both CMV and *T. gondii* IgG Vidas ELFAs, we found that 75 % (59/79) of the samples were positive in the CMV IgG SIA while 60 % (46/77) were positive in the *T. gondii* IgG SIA (Fig. 3).

Compared to Vidas ELFAs, the multiplex CMV and *T. gondii* IgG SIAs showed 100 % sensitivity and specificity; with very good agreement (kappa coefficient with 95 %; CI: 1).

The intra-assay and inter-assay variability of CMV IgG SIA were 3 ~ 7 % and 4 ~ 9 %, and those of *T. gondii* IgG SIA were 7 ~ 12 % and 8 ~ 15 %, respectively.

### Multiplex IgG SIA

**Fig. 4:**
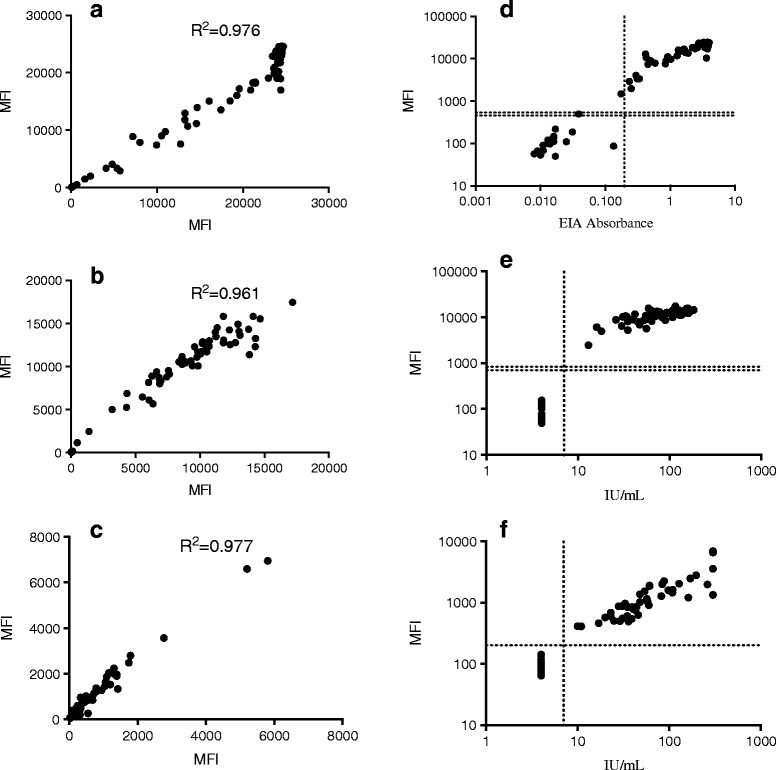
Comparison of anti-B19V, −CMV and -*T. gondii* IgG seroresponses by multiplex and singleplex SIAs/EIA. The left figures (**a**), (**b**) and (**c**) represented B19, CMV and *T. gondii* IgG responses by multiplex SIAs (*y-axis*) and singleplex SIAs (*x-axis*), respectively. The right figures (**d**), (**e**) and (**f**) represented B19V, CMV and *T. gondii* IgG responses by multiplex SIAs (*y-axis*) and the corresponding reference EIA/ELFA *(x-axis),* respectively. Horizontal dot lines indicate the cutoffs between negative and positive samples

We tested the B19V, CMV and *T. gondii* IgG SIAs with 80 sera of known IgG reactivity in the multiplex format. Pearson correlation coeffients (R^2^) in Fig. 4 shows good correlation between multiplex and singleplex SIA. The B19V IgG seropositivity corresponded well with those obtained with EIA except for a single discrepancy (SIA+, EIA-). The CMV and T. gondii IgG seropositivity corresponded perfectly with those obtained with Vidas ELFA, though the assay condition as specified for the B19V IgG SIA. The good performance of the multiplex IgG SIA indicated no interference among the three IgG SIAs (Fig. 4).

The intra-assay and inter-assay variability of multiplex B19V IgG SIA were 2 ~ 8 % and 1 ~ 9 %, of multiplex CMV IgG SIA were 4 ~ 8 % and 5 ~ 13 %, of multiplex *T. gondii* IgG SIA were 6 ~ 9 % and 8 ~ 12 %, respectively.

## Discussion

We established and validated singleplex and multiplex microsphere-based platforms for simultaneous detection of IgG against B19V, *T. gondii* and CMV. The assays were highly sensitive and specific, with no interference observable. All assays had a wide dynamic range, albeit with *T. gondii* lower than with B19V or CMV. The CMV and *T. gondii* IgG SIAs performed well in the multiplex format, using the same assay conditions as for the B19V IgG SIA e.g. magnetic microspheres, indicating good assay reproducibility.

The SIAs detected reliably the seroresponses against these three pathogens with an estimated net cost per sample of 0.2€ [13] for singleplex and 0.35€ for triplex, making these tools beneficial financially. Moreover, multiplex is superior to singleplex in terms of time, sample volume, amount of reagents and hands-on time. The multiplex- Luminex technology is flexible, and other assays maybe included in the present panel.

It also allows the possibility of including internal controls to individual samples thus providing a measure of the reliability of each readout.

Our SIA results were highly concordant with those of other immunoassays of various types (in-house EIA, Biotrin’s EIA, Vidas ELFA). The few discrepancies noted, occurred mostly in samples from early infection, possibly due to low antibody affinity. Other explanations may lay in the intrinsic differences between SIA and EIA-based reference assays [14, 15]: such as (i) fluorescence vs. colorimetric substrate detection, (ii) antigen coupling covalently onto magnetic microspheres vs. via adsorption onto streptavidin-coated plates in our in house EIAs, (iii) antibody detection with protein G as opposed to anti-human IgG.

Of note, during the development of these assays, we found that post-coat blocking of the microsphere with bovine serum albumin (BSA) yielded inappropriately high background in some individuals (our unpublished data). However, replacement of BSA with Tris significantly improved the signal-to-noise ratios [10].

In this study, we used well-characterised cohorts of sera and controls in our singleplex and multiplex SIAs and we show good reproducibility in comparison to reference assays. However, as the test parameters were calculated based on a limited number of samples, its performance should be further validated with other clinical cohorts. Moreover, other assays such as IgG-avidity and IgM may aid in the diagnosis of acute infection and are currently under development in our lab.

## Conclusion

We showed that IgG antibodies against B19V, *T. gondii* and CMV are successfully detected using the microsphere-based suspension assay both in single and multiplex formats, making it a powerful microbiological tool.

## Additional file

Additional file 1:
**Cost calculation.** (DOCX 82 kb)

## Abbreviations

B19V: Human parvovirus B19
BSA: Bovine serum albumin
CMV: Cytomegalovirus
EIA: Enzyme immunoassay
ELFA: Enzyme linked fluorescent assay
MFI: Median fluorescence intensity
PBST: Phosphate buffered saline with 0.05 % Tween-20
SG: StabilGuard
SIA: Suspension immuno assay
*T. gondii*: *Toxoplasma gondii*
VLP: Virus like particle
