# Supplementary material for: Microsphere-based antibody assays for human parvovirus B19V, CMV and T. gondii
Source: BMC Infect Dis. 2016 Jan 8;16:8. doi: 10.1186/s12879-015-1194-3 (PMC4706663; doi:10.1186/s12879-015-1194-3)
Supplement: Additional file 1: — Cost calculation. (DOCX 82 kb) [file 12879_2015_1194_MOESM1_ESM.docx]

| Item | Price/well | | |
| --- | --- | --- | --- |
|  | Singleplex | Duplex | Triplex |
| Magnetic COOH Beads | 0.05 € | 0.1€ | 0.15 € |
| Protein G | 0.04 € | 0.04€ | 0.04 € |
| Streptavidin-phycoerythrin | 0.06 € | 0.06€ | 0.06 € |
| Wash buffer | 0.03 € | 0.03€ | 0.03 € |
| 96-well Plate | 0.01 € | 0.01€ | 0.01 € |
| Others (buffer, etc.) | 0.02 € | 0.02 € | 0.02 € |
| Antigen  (In-house) | 0.008 € | 0.016€ | 0.024€ |
| Antigen  (Commercial) | 0.06 € | 0.12 € | 0.18 € |
| Total  (In-house antigen) | 0.21 € | 0.28 € | 0.35 € |
| Total  (Commercial antigen) | 0.27 € | 0.38 € | 0.44 € |

**Cost Calculation**
